# Supplementary material for: Chemical Fingerprinting of Heat Stress Responses in the Leaves of Common Wheat by Fourier Transform Infrared Spectroscopy
Source: Int J Mol Sci. 2022 Mar 4;23(5):2842. doi: 10.3390/ijms23052842 (PMC8911002; doi:10.3390/ijms23052842)
Supplement: Supplementary file 1 [file ijms-23-02842-s001.zip › Supplementary File S1.pdf]

Supplementary File S1.
Osman *et. al.*
R-scripts for the processing of FT-IR data

<u>Script code 1: FTIR-spectra processing</u>

```
#salma_a2_spec_processing_ftir_211213.r
#import necessary libraries
library(conflicted)
library(dplyr)
library(ggplot2)
library(readr)

#clean up the R's brain
rm(list=ls())

#obtain date information
today <- Sys.Date()
yr <- substr(today, 3,4)
mo <- substr(today, 6,7)
day <- substr(today, 9,10)
today2 <- paste(yr, mo, day, sep="")

#obtain desktop folder information for the windows user
#change the string within "xxx" below according to your computer
desktopfolder <- "akash"

#create column names for output dataframe
wnlist <- seq(4000, 400, length=3601)
columnname <- c("filename", "condition", "genotype", "identifier", wnlist)

#name the column labels for spec data
specpile <- as.data.frame(t(columnname))
names(specpile) <- columnname
specpile <- slice(specpile, -1)

#set working directory
```

```
#setwd("C:/Users/akash/desktop/inputfolder")

#input data from .asc file that are generated by Perkin-Elmer
#obtain the filename
#obtain the list of filenames for all csv files,
  #which are transiently stored in "ftir_spec_input" folder in your desktop
pathname_inputfolder  <-  paste("C:/Users/",desktopfolder,  "/desktop/",  "ftir_spec_input",
sep="")
filelist <- list.files(path = pathname_inputfolder,
                       pattern = "*.asc",
                       full.names = T)

#count the number of files
fileno <- length(filelist)

#starting a loop for processing data
for (i in 1:fileno){

 #obtain the new filename
    filename <- basename(filelist[i])

 #obtain dataframe
 #skip first 25 lines
 #the 26th line does not have variable names
  rawspec <- read.table(filelist[i], skip = 25)

 #quick summary
#   summary(rawspec)

 #plot the spectrum
#   ggplot(rawspec, aes(x = V1,y = V2)) +
#     geom_point()

 #save the wn column for later plotting
  wn_column <- dplyr::select(rawspec, V1)
```

```
#exchange rows and columns
#(optional)keep the type as data.frame
  rawspec2 <- as.data.frame(t(rawspec))

#split the rows into wn and spec
  wn_axis <- as.data.frame(rawspec2[1,])
  rawspec3 <- as.data.frame(rawspec2[2,])

#name the column labels for spec data
  names(rawspec3) <- wn_axis

  #smoothing of the spectrum trace
  #below to fill in, but currently skip it

  #obtain the baseline anchors
  #this is a version to take only 4000 and 400
  #the relationships between wn and column_no. is
  #column_no = -wn +4001
  raw400 <- rawspec3[1,3601]
  raw4000 <- rawspec3[1,1]

  #create a baseline data
  #following is the 1st version
  #line is drawn between 4000 to 400
  baseline <- seq(raw4000, raw400, length=3601)

  #subtract the baseline
  spec4_baselined <- (rawspec3 - baseline)

  #draw the baseline-corrected spectrum
  #1st, exchange the rows and columns
  spec4_tall <- t(spec4_baselined)

  #combine the wn and spec columns
  spec4_tall <- cbind(wn_column, spec4_tall)
```

```
#plot the baseline-corrected spectrum
#ggplot(spec4_tall, aes(x = V1,y = V2)) +
#    geom_point(size=0.3)

#normalization of spec
#1st, sum of current spec is calculated
sum_signal_original <- sum(select(spec4_tall, V2))

#2nd, new column is generated in the spec
#spec values in ppm is calculated
spec5_tall <- dplyr::mutate(spec4_tall, ABS = V2*1000000/sum_signal_original)

#draw the normalized spectrum
#ggplot(spec5_tall, aes(x = V1,y = ABS)) +
#    geom_point(size=0.3)

#row-column conversion
spec5 <- as.data.frame(t(spec5_tall))

#remove original data from spec5
spec6 <- dplyr::slice(spec5, 3)

#rownames(spec6) <- filename

#create one column at the top
#add dataname to the 1st column
spec7 <-    mutate(spec6, dataname=filename, .before="4000")

#judge the treatment condition
#and add to the 2nd column
condition_id <- substring(filename, 1, 2)
spec8 <- mutate(spec7, condition=condition_id, .after="dataname")

#judge the genotype
#and add to the 3rd column
genotype_id <- substring(filename, 3, 5)
```

```
  spec9 <- mutate(spec8, genotype=genotype_id, .after="condition")

  #setup the identifier for later analyses
  #and add to the 4rd column
  identifier_column <- substring(filename, 1, 5)
  spec10 <- mutate(spec9, identifier=identifier_column, .after="genotype")

  #compiling the data
  specpile <- rbind(specpile, spec10)
  }

#export the data as csv
#data is baseline-corrected, normalized spec
filename_specpile_processed <- paste(today2, "_", "specpile_processed.csv", sep="")
filename2_specpile_processed <- paste("C:/users/",desktopfolder,"/desktop/",
filename_specpile_processed, sep="")
write.csv(specpile,
          filename2_specpile_processed, row.names=FALSE)

#prepare long-format as well, and export
#row-column conversion
long_specpile <- as.data.frame(t(specpile))

#create new column at the top
long_specpile <- mutate(long_specpile,
                        variable=c("dataname","condition", "genotype", "identifier",
                                   seq(from=4000, to=400, by=-1)),
                    .before=ABS)

#export the data as csv
#data is baseline-corrected, normalized spec
filename_specpile_processed_longformat <- paste(today2, "_",
"specpile_processed_longformat.csv", sep="")
filename2_specpile_processed_longformat <- paste("C:/users/",desktopfolder,"/desktop/",
filename_specpile_processed_longformat, sep="")
write.csv(long_specpile,
```

```
        filename2_specpile_processed_longformat, row.names=FALSE)
#End of script
```

Script code 2: Principal component analysis

```
#salma_a3_pca_ftir_211213.r
#ftir_PCA for salma's paper 1
#this is a version to analyze differences in c3-h3 samples

#imput file should be in csv format,
#typically, "specpile_processed.csv" would be selected
#1st column should be the names of original spec files
#2nd col should be treatment ID such as c0, h3, c3
#3rd col should be genotype such as n61, ima,
#4th col should be "identifier" such as c0n61, which is used for grouping
#then followed by abs values from 4000 to 400

#import necessary libraries
library(conflicted)
library(dplyr)
library(ggplot2)
library(readr)
library(psych)

#clean up the R's brain
rm(list=ls())

#obtain desktop folder information for a windows user
#you must change the string within "xxx" below according to your computer
desktopfolder <- "akash"

#obtain date information
today <- Sys.Date()
yr <- substr(today, 3,4)
mo <- substr(today, 6,7)
day <- substr(today, 9,10)
```

```
today2 <- paste(yr, mo, day, sep="")

#invoke a file-opening window, specify the file,
#input data from .csv file
#data has to be baseline-corrected and normalized
#obtain the filename
inputfile <- file.choose()
filename <- basename(inputfile)
rawspecs <- read.csv(inputfile,
                     header = T)
filename

#extract values used for calculation to a new df specmatrix
#values for wn4000 and 400 (zero values) should be removed
specmatrix <- dplyr::select(rawspecs, -(3605:3605))
specmatrix <- dplyr::select(specmatrix, -(1:5))

#separate "identifier" column (category info)
id_1 <- dplyr::select(rawspecs, (4:4))

#further trimming of values in the range of wn3600-4000
specmatrix <- dplyr::select(specmatrix, -(1:399))

#perform pca analysis using prcomp
#prcomp is standard but one of the oldest
pc = prcomp(specmatrix, scale =T)

#using the new 'principal()' in psych package
#pc <- psych::principal(specmatrix, nfactors=3601,
#                       rotate='none')

#display the summary
summary(pc)

#preparation of score data output
pc1_score <- pc$x[,1]
```

```
pc2_score <- pc$x[,2]
scoreonly <- as.data.frame(pc$x)
score <- cbind(id_1, scoreonly)

#export the score data as csv
#data is PC1-PC2 score
filename_PC12_score <- paste(today2, "_", "PC12_score.csv", sep="")
filename2_PC12_score <- paste("C:/users/",desktopfolder,"/desktop/",
                              filename_PC12_score, sep="")
write.csv(score,
        filename2_PC12_score, row.names=FALSE)



#export rotation data as csv file
rotationonly <- as.data.frame(pc$rotation)
filename_pca_rotation <- paste(today2, "_", "pca_rotation.csv", sep="")
filename2_pca_rotation <- paste("C:/users/",desktopfolder,"/desktop/",
                              filename_pca_rotation, sep="")
write.csv(rotationonly,
        filename2_pca_rotation, row.names=FALSE)

#export sdev data as csv file
sdevonly <- as.data.frame(pc$sdev)
filename_pca_sdev <- paste(today2, "_", "pca_sdev.csv", sep="")
filename2_pca_sdev <- paste("C:/users/",desktopfolder,"/desktop/",
                              filename_pca_sdev, sep="")
write.csv(rotationonly,
        filename2_pca_sdev, row.names=FALSE)

#calculate the loadings, and export as csv file
loadingdata <- sweep(pc$rotation, MARGIN=2, pc$sdev, FUN="*")
filename_pca_loading <- paste(today2, "_", "pca_loading.csv", sep="")
filename2_pca_loading <- paste("C:/users/",desktopfolder,"/desktop/",
                              filename_pca_loading, sep="")
write.csv(loadingdata,
        filename2_pca_loading, row.names=FALSE)
```

```
#draw the pc1_pc2 scoreplot
#size of the dots in the plot can be changed
   #by modifying the location of "geom_point(size=)
dev.new()
pca_scoreplot <- ggplot(score, aes(x = PC1,y = PC2,
                                                       color = identifier)) +
   geom_point(size=2) +
   scale_color_manual(values=c("deepskyblue", "salmon")) +
   theme_bw()
print(pca_scoreplot)

#save the plot as png format
#you can change to .jpeg, .tiff, etc
#unit is in inch
filename_pca_scoreplot <- paste(today2, "_", "pca_scoreplot.csv", sep="")
filename2_pca_scoreplot <- paste("C:/users/",desktopfolder,"/desktop/",
                                                    filename_pca_scoreplot, ".png", sep="")
ggsave(file = filename2_pca_scoreplot,
          plot = pca_scoreplot, dpi=100,
          width=7.2, height=4.8)

#extract contribution data
contribution <- as.data.frame(t(summary(pc)$importance))
names(contribution)                                                                                        <-
c("standard_deviation","proportion_of_variance","cumulative_proportion")
contri_rownames <- as.data.frame(rownames(contribution))
names(contri_rownames) <- "PC"
contribution <- cbind(contribution, contri_rownames)

#save the contribution data as csv file
#write.csv(contribution, "C:/Users/akash/desktop/contribution.csv")

filename_pca_contribution <- paste(today2, "_", "pca_contribution.csv", sep="")
filename2_pca_contribution <- paste("C:/users/",desktopfolder,"/desktop/",
                                                 filename_pca_contribution, sep="")
```

```r
write.csv(contribution,
          filename2_pca_contribution, row.names=FALSE)

#extract top 9 from the contribution data, and save as a png file
top9_contribution <- dplyr::slice(contribution, 1:9)

contribution_plot <- ggplot(top9_contribution, aes(x = PC, y = proportion_of_variance)) +
  geom_bar(stat="identity", fill="forestgreen") +
  theme_bw()
print(contribution_plot)
#ggsave(file = "C:/Users/akash/desktop/contribution_plot.png",
#        plot = contribution_plot, dpi = 100,
#        width = 3.6, height = 2.4)

filename_pca_contribution_plot <- paste(today2, "_", "pca_contribution_plot.csv", sep="")
filename2_pca_contribution_plot <- paste("C:/users/",desktopfolder,"/desktop/",
                                         filename_pca_contribution_plot, ".png", sep="")
ggsave(file = filename2_pca_contribution_plot,
       plot = contribution_plot, dpi=100,
       width=7.2, height=4.8)
#End of script
```

Script code 3: Linear Discriminant Analysis

```r
#salma_a4_lda_ftir_211220.r
#a4_lda_ftir
  #linear discriminant analysis of ftir spectra

#clear the brain
rm(list=ls())

#library to register
#ggplot2 and dplyr are in tidyverse
library(conflicted)
library(tidyverse)
library(MASS)
```

```
library(klaR)
library(caret)

#obtain date information
today <- Sys.Date()
yr <- substr(today, 3,4)
mo <- substr(today, 6,7)
day <- substr(today, 9,10)
today2 <- paste(yr, mo, day, sep="")

#obtain desktop folder information for a windows user
#you must change the string within "xxx" below according to your computer
desktopfolder <- "akash"

#assemble path info
pathinfo <- paste("C:/users/",desktopfolder,"/desktop/", sep="")

#import the compiled ftir csv data
#the file to choose is normally "ftir_specpile_processed.csv"
spec1 <- file.choose()
spec2 <- read.csv(spec1,
                  header = T)

#remove genotype and dataname info
specmatrix <- dplyr::select(spec2, -(1:3))

#remove values at wavenumbers 4000 and 400,
#which were used for baseline anchors
#then remove those at 3999-3601, the noisy region
specmatrix <- dplyr::select(specmatrix, -3602)
specmatrix <- dplyr::select(specmatrix, -2)
specmatrix <- dplyr::select(specmatrix, -(2:400))

#treatment <- dplyr::select(rawspecs, (2:2))

#set the seednumber for randomness
```

```
set.seed(101)

#split the samples into train(60%) and test(40%)
training_sample <- sample(c(TRUE, FALSE), nrow(specmatrix), replace = T, prob = c(0.6,
0.4))
trainspec <- specmatrix[training_sample, ]
testspec <- specmatrix[!training_sample, ]

#perform linear discriminant analysis
lda_spec_train <- lda(identifier ~ ., trainspec)
lda_spec_train

#training results check
#1st, transform them to the values
#then one dimensional histograms
#"mar" is the margin of bottom, left, top, right
lda_spec_train_results <- predict(lda_spec_train)
dev.new()
par("mar"=c(1,1,1,1))
trainhistogram1 <- ldahist(lda_spec_train_results$x[,1], g=trainspec$identifier)
print(trainhistogram1)

#save the histogram values for the training set as csv file
class_lda_spec_train <- as.data.frame(lda_spec_train_results$class)
x1_lda_spec_train <- as.data.frame(lda_spec_train_results$x[,1])
value_lda_spec_train <- cbind(class_lda_spec_train, x1_lda_spec_train)
names(value_lda_spec_train) <- c("identifier", "LD1")
filename_value_lda_spec_train <- paste(today2, "_", "value_lda_spec_train.csv", sep="")
filename2_value_lda_spec_train    <-    paste(pathinfo,"/",    filename_value_lda_spec_train,
sep="")
write.csv(value_lda_spec_train,
          filename2_value_lda_spec_train, row.names=FALSE)

#draw histogram of train results for publication
dev.new()
lda_train_histogram2 <- ggplot(value_lda_spec_train,
```

```
                                             aes(x = LD1, fill =identifier)) +
   geom_histogram(position="identity",
                          colour = "black", size=0.3,
                          breaks=seq(from=-80, to=60, by=2)) +
   scale_fill_manual(values=c("deepskyblue", "salmon")) +
   theme_bw()
print(lda_train_histogram2)

#save the plot as png format
#you should change the path according to your system
#you can change to .jpeg, .tiff, etc
#unit is in inch
filename_lda_train_histogram2 <- paste(today2, "_", "lda_train_histogram2.png", sep="")
filename2_lda_train_histogram2 <- paste(pathinfo,"/", filename_lda_train_histogram2,
sep="")
ggsave(file = filename2_lda_train_histogram2,
            plot = lda_train_histogram2, dpi=100,
            width=7.2, height=3.6)

#test set check
#1st, transform them to the values
#then one dimensional histograms
lda_spec_test_results <- predict(lda_spec_train, testspec)
dev.new()
par("mar"=c(1,1,1,1))
testhistogram1 <- ldahist(lda_spec_test_results$x[,1], g=testspec$identifier)
print(testhistogram1)

#save the histogram values for the test set as csv file
class_lda_spec_test <- as.data.frame(lda_spec_test_results$class)
x1_lda_spec_test <- as.data.frame(lda_spec_test_results$x[,1])
value_lda_spec_test <- cbind(class_lda_spec_test, x1_lda_spec_test)
names(value_lda_spec_test) <- c("identifier", "LD1")
filename_value_lda_spec_test <- paste(today2, "_", "value_lda_spec_test.csv", sep="")
filename2_value_lda_spec_test <- paste(pathinfo,"/", filename_value_lda_spec_test, sep="")
write.csv(value_lda_spec_test,
```

```
                filename2_value_lda_spec_test, row.names=FALSE)

#draw histogram of test results for publication
dev.new()
lda_test_histogram2 <- ggplot(value_lda_spec_test,
                                              aes(x = LD1, fill =identifier)) +
  geom_histogram(position="identity",
                      colour = "black", size=0.3,
                      breaks=seq(from=-80, to=60, by=2)) +
  scale_fill_manual(values=c("deepskyblue", "salmon")) +
  theme_bw()
print(lda_test_histogram2)

#save the plot as png format
#you should change the path according to your system
#you can change to .jpeg, .tiff, etc
#unit is in inch
filename_lda_test_histogram2 <- paste(today2, "_", "lda_test_histogram2.png", sep="")
filename2_lda_test_histogram2 <- paste(pathinfo,"/", filename_lda_test_histogram2,
sep="")
ggsave(file = filename2_lda_test_histogram2,
          plot = lda_test_histogram2, dpi=100,
          width=7.2, height=3.6)

#extract LD1 loading
scalingdata <- lda_spec_train$scaling

#transform LD1 loading to dataframe
#add wavenumber info
scalingdf <- as.data.frame(t(scalingdata))
wnlist <- seq(3600, 401, length=3200)
wnlist2 <- as.data.frame(t(wnlist))

colnames(scalingdf) <- c(seq(3600, 401, length=3200))
colnames(wnlist2) <- c(seq(3600, 401, length=3200))
```

```
scalingdf2 <- rbind(wnlist2, scalingdf)
scalingdf3 <- as.data.frame(t(scalingdf2))
names(scalingdf3)[1] <- "wavenumber"

#change the wavenumber in ascending order, and save it as csv
scalingdf4 <- arrange(scalingdf3, wavenumber)
filename_scalingdf4 <- paste(today2, "_", "LD1_loading.csv", sep="")
filename2_scalingdf4 <- paste(pathinfo,"/", filename_scalingdf4, sep="")
write.csv(scalingdf4,
          filename2_scalingdf4, row.names=FALSE)

#plot LD1 contribution, scatter plot version
dev.new()
lda_loading_scatterplot <- ggplot(scalingdf4, aes(x = wavenumber, y = LD1)) +
  geom_point(size=0.5) +
  theme_bw()
print(lda_loading_scatterplot)

filename_lda_loadingscatterplot <- paste(today2, "_", "LDA_Loading_ScatterPlot.png",
sep="")
filename2_lda_loadingscatterplot <- paste(pathinfo,"/", filename_lda_loadingscatterplot,
".png", sep="")
ggsave(file = filename2_lda_loadingscatterplot,
       plot = lda_loading_scatterplot, dpi = 100,
       width = 7.2, height = 4.8)

#plot LD1 contribution, line plot version
dev.new()
lda_loading_lineplot <- ggplot(scalingdf4, aes(x=wavenumber, y=LD1))+
  geom_line(size=0.2)+
  theme_bw()
print(lda_loading_lineplot)

filename_lda_loadinglineplot <- paste(today2, "_", "LDA_Loading_LinePlot.png", sep="")
filename2_lda_loadinglineplot <- paste(pathinfo,"/", filename_lda_loadinglineplot, ".png",
sep="")
```

```
ggsave(file = filename2_lda_loadinglineplot,
       plot = lda_loading_lineplot, dpi = 100,
       width = 7.2, height = 4.8)

#pick up peak candidate in LD1 plot
#that are higher than the threshold of 0.15
peakcandidate1 <- dplyr::filter(scalingdf4, LD1>0.15)

#check that the candidate is the higher than the neighboring wavenumbers
ncandidate <- nrow(peakcandidate1)
peakcandidate2 <- data.frame(matrix(rep(NA,8),nrow=1))[numeric(0),]
colnames(peakcandidate2) <-
  c("wavenumber","LD1","LD1m1","LD1p1","peak",    "GoFurther","LargerWnBoundary",
"SmallerWnBoundary")

for(i in 1:ncandidate){
  peakcandidate_tempo <- data.frame(matrix(rep(NA,8),nrow=1))[numeric(0),]
  colnames(peakcandidate_tempo) <-
    c("wavenumber","LD1","LD1m1","LD1p1","peak","GoFurther","LargerWnBoundary",
"SmallerWnBoundary")
  wn_quest <- peakcandidate1[i,1]
  wn_quest_m1 <- wn_quest - 1
  wn_quest_p1 <- wn_quest + 1
  peakcandidate_tempo[1,1] <- wn_quest
  peakcandidate_tempo[1,2] <- scalingdf4[wn_quest-400,2]
  peakcandidate_tempo[1,3] <- scalingdf4[wn_quest_m1-400,2]
  peakcandidate_tempo[1,4] <- scalingdf4[wn_quest_p1-400,2]
  if(peakcandidate_tempo[1,2]>peakcandidate_tempo[1,3]
     & peakcandidate_tempo[1,2]>peakcandidate_tempo[1,4]){
    peakcandidate_tempo[1,5] <- 1
  }
  peakcandidate2 <- rbind(peakcandidate2, peakcandidate_tempo)
}

#save the peak candidate as csv file
#change the wavenumber in descending order of LD1, and save it as csv
```

```
peakcandidate3 <- dplyr::filter(peakcandidate2, peak==1)
peakcandidate3 <- arrange(peakcandidate3, desc(LD1))
filename_peakcandidate <- paste(today2, "_", "PeakCandidateList.csv", sep="")
filename2_peakcandidate <- paste(pathinfo,"/", filename_peakcandidate, sep="")
write.csv(peakcandidate3,
          filename2_peakcandidate, row.names=FALSE)

#pick up valley candidate in LD1 plot
#that are lower than the threshold of -0.15
valleycandidate1 <- dplyr::filter(scalingdf4, LD1 < -0.15)

#check that the candidate is the higher than the neighboring wavenumbers
n_valleycandidate <- nrow(valleycandidate1)
valleycandidate2 <- data.frame(matrix(rep(NA,8),nrow=1))[numeric(0),]
colnames(valleycandidate2) <-
  c("wavenumber","LD1","LD1m1","LD1p1","valley","GoFurther","LargerWnBoundary",
"SmallerWnBoundary")

for(i in 1:n_valleycandidate){
  valleycandidate_tempo <- data.frame(matrix(rep(NA,8),nrow=1))[numeric(0),]
  colnames(valleycandidate_tempo) <-
    c("wavenumber","LD1","LD1m1","LD1p1","valley","GoFurther","LargerWnBoundary",
"SmallerWnBoundary")
  wn_quest <- valleycandidate1[i,1]
  wn_quest_m1 <- wn_quest - 1
  wn_quest_p1 <- wn_quest + 1
  valleycandidate_tempo[1,1] <- wn_quest
  valleycandidate_tempo[1,2] <- scalingdf4[wn_quest-400,2]
  valleycandidate_tempo[1,3] <- scalingdf4[wn_quest_m1-400,2]
  valleycandidate_tempo[1,4] <- scalingdf4[wn_quest_p1-400,2]
  if(valleycandidate_tempo[1,2] < valleycandidate_tempo[1,3]
     & valleycandidate_tempo[1,2] < valleycandidate_tempo[1,4]){
    valleycandidate_tempo[1,5] <- 1
  }
  valleycandidate2 <- rbind(valleycandidate2, valleycandidate_tempo)
}
```

```
#save the valley candidate as csv file
#change the wavenumber in ascending order of LD1, and save it as csv
valleycandidate3 <- dplyr::filter(valleycandidate2, valley==1)
valleycandidate3 <- arrange(valleycandidate3, LD1)
filename_valleycandidate <- paste(today2, "_", "ValleyCandidateList.csv", sep="")
filename2_valleycandidate <- paste(pathinfo,"/", filename_valleycandidate, sep="")
write.csv(valleycandidate3,
          filename2_valleycandidate, row.names=FALSE)
#End of script
```

Script code 4: Quest anchors for Fm-markers

```
#salma_a5_quest_anchors_lda_211220.r
#quest anchors for LDA peaks
  #for identifying the most effective pair of anchor points
  #that show peaks in LDA contribution plot
#this is for Salma's data on N61 c3-h3 chamber comparison.

#clear the brain
rm(list=ls())

#library to register
#ggplot2 and dplyr are in tidyverse
library(conflicted)
library(tidyverse)
library(MASS)
library(klaR)
library(caret)

#obtain date information
today <- Sys.Date()
yr <- substr(today, 3,4)
mo <- substr(today, 6,7)
day <- substr(today, 9,10)
today2 <- paste(yr, mo, day, sep="")
```

```
#obtain desktop folder information for a windows user
#you must change the string within "xxx" below according to your computer
desktopfolder <- "akash"

#assemble path info
pathinfo <- paste("C:/users/",desktopfolder,"/desktop/", sep="")

#import a "xxxxxx_PeakCandidateXXXX.csv"
#that is modified from PeakCandidateList2.csv
#the import file is a dataframe, and it contains following 8 columns
#"wavenumber, LD1, LD1m1, LD1p1, peak, GoFurther, LargerWnBoundary, SmallerWnBoundary"
#1st row is the column name, and data is in 2nd row
#the last two typically set at 150 larger and smaller than the target wavenumber
print("Please specify xxxxxx_a4_PeakCandidateXXXX.csv")
PeakCandidateInput <- file.choose()
PeakCandidateInput2 <- read.csv(PeakCandidateInput,
                                header = T)

#import LDA-peak and boundary information
lda_peak_wn <- PeakCandidateInput2[1,1]
lda_LargerWnBoundary_wn <- PeakCandidateInput2[1,7]
lda_SmallerWnBoundary_wn <- PeakCandidateInput2[1,8]

#modify peak_wn variable to the style of column name
lda_peak_wn_string <- as.character(lda_peak_wn)
lda_peak_wn_colname <- paste("X",lda_peak_wn_string, sep="")

#import the compiled ftir csv data
#the file to choose is normally "a2_specpile_processed.csv"
print("Please specify xxxxxx_a2_specpile_processed.csv")
spec1 <- file.choose()
specmatrix <- read.csv(spec1,
                       header = T)
```

```
#separate the data into c3 and h3
c3matrix <- dplyr::filter(specmatrix, identifier == "c3n61")
h3matrix <- dplyr::filter(specmatrix, identifier == "h3n61")

#now, the matrix data should have 223 columns
#consist of first 2 factorial data, followed by 221 wn data columns
#sum of wn and column number equals to 663
#thus, wn576 correspond to column 87.
#extract the wn576 data from each group

c3peakabs <- dplyr::select(c3matrix, lda_peak_wn_colname)
h3peakabs <- dplyr::select(h3matrix, lda_peak_wn_colname)
totalpeakabs <- dplyr::select(specmatrix, lda_peak_wn_colname)

#2: An empty output dataframe generated.
#rep(NA, 27) is a function to generate NA for 47 times.
quest_anchor_summary <- data.frame(matrix(rep(NA, 47), nrow=1))[numeric(0), ]
colnames(quest_anchor_summary) <- c("wn1and2", "wn1", "wn2",
   "h3a_HigherMedian", "h3b_HigherMedian", "a_h_c_ratio", "b_h_c_ratio", "p_a", "p_b",
   "hih3_a_score_boxplot", "hih3_b_score_boxplot",
   "loh3_a_score_boxplot", "loh3_b_score_boxplot",
   "sand_score_a", "sand_score_b",
   "hih3_a_BoxSeparated", "hih3_c3a_med_under_h3a1stQ",
"hih3_c3a3rdQ_under_h3a_med",
   "hih3_b_BoxSeparated", "hih3_c3b_med_under_h3b1stQ",
"hih3_c3b3rdQ_under_h3b_med",
   "loh3_a_BoxSeparated", "loh3_c3a_med_over_h3a3rdQ", "loh3_c3a1stQ_over_h3a_med",
   "loh3_b_BoxSeparated", "loh3_c3b_med_over_h3b3rdQ", "loh3_c3b1stQ_over_h3b_med",
   "sand_c3a_median", "sand_c3a_1stQ", "sand_h3a_median", "sand_h3a_1stQ",
   "sand_c3b_median", "sand_c3b_1stQ", "sand_h3b_median", "sand_h3b_1stQ",
   "c3a_1stQ", "c3a_median", "c3a_3rdQ", "h3a_1stQ", "h3a_median", "h3a_3rdQ",
   "c3b_1stQ", "c3b_median", "c3b_3rdQ", "h3b_1stQ", "h3b_median", "h3b_3rdQ")

#3: Outward looping start
#loop should be from 100-higher wn from the peak, i.e., lda_LargerWnBoundary_wn,
#to 10-higher wn from the peak
```

```
#loop value is specified by column number, i.e., wn4000 is in col5, wn3999 is in col6
   #the sum of wn"xxxx" and col"x" is 4005.
   #thus, col number for lda_LargerWnBoundary_wn should be "4005-lda_LargerWnBoundary_wn
   #col number for lda_peak_wn is "4005-Lda_peak_wn"
   #col number for 10-higher wn from the peak is "3995-lda_peak_wn"
#modify the line below to
   #"i_startpoint:i_startpoint" for pilot test,
   #and "i_startpoint:i_endpoint" for full calculation
#for (i in i_startpoint:i_endpoint){
i_startpoint <- 4005 - lda_LargerWnBoundary_wn
i_endpoint <- 3995 - lda_peak_wn
for (i in i_startpoint:i_endpoint){

      #4: Setting the anchor1 value.
   c3anchor1 <- dplyr::select(c3matrix, i)
   h3anchor1 <- dplyr::select(h3matrix, i)
   anchor1total <- dplyr::select(specmatrix, i)

   #5: Inward looping start.
   #loop should be from 10-step downstream from the peak,
      #col number for lda_peak_wn is "4005-Lda_peak_wn"
      #thus col number for 10-step downstream is "4015-lda_peak_wn"
   #the loop is stopped at "lda_SmallerWnBoundary_wn"
      #col number for "lda_SmallerWnBoundary_wn" is "4005-lda_SmallerWnBoundary_wn"
   #modify the line below to
         #"j_startpoint:j_startpoint" for pilot test,
      #and "j_startpoint:j_endpoint" for full calculation
   #for (j in (j_startpoint:j_endpoint)){
   j_startpoint <- 4015 - lda_peak_wn
   j_endpoint <- 4005 - lda_SmallerWnBoundary_wn
   for (j in j_startpoint:j_endpoint){
      c3anchor2 <- dplyr::select(c3matrix, j)
      h3anchor2 <- dplyr::select(h3matrix, j)
      anchor2total <- dplyr::select(specmatrix, j)
```

```
#set up a temporary df for the results
tempo_anchor_results <- data.frame(matrix(rep(NA, 47), nrow=1))[numeric(0), ]
tempo_candidate_hih3_wn1base <- data.frame(matrix(rep(NA, 44), nrow=1))[numeric(0), ]
tempo_candidate_hih3_wn2base <- data.frame(matrix(rep(NA, 44), nrow=1))[numeric(0), ]
tempo_candidate_loh3_wn1base <- data.frame(matrix(rep(NA, 44), nrow=1))[numeric(0), ]
tempo_candidate_loh3_wn2base <- data.frame(matrix(rep(NA, 44), nrow=1))[numeric(0), ]

#record the anchors info
wn1 <- colnames(specmatrix)[i]
wn2 <- colnames(specmatrix)[j]
wn1and2 <- paste(as.character(wn1), as.character(wn2), sep="and")

#define formula for ftir-marker(fm)
#set the wn1 as basepoint, calculate marker "a" value
#set the wn2 as basepoint, calculate marker "b" value
c3fma <- (c3peakabs-c3anchor1)/(c3anchor2-c3anchor1)
h3fma <- (h3peakabs-h3anchor1)/(h3anchor2-h3anchor1)
c3fmb <- (c3peakabs-c3anchor2)/(c3anchor1-c3anchor2)
h3fmb <- (h3peakabs-h3anchor2)/(h3anchor1-h3anchor2)

#6: Calculate the key statistics
c3a_summary <- summary(c3fma[,1])
c3a_1stQ <- as.numeric(c3a_summary)[2]
c3a_median <- as.numeric(c3a_summary)[3]
c3a_3rdQ <- as.numeric(c3a_summary)[5]
h3a_summary <- summary(h3fma[,1])
h3a_1stQ <- as.numeric(h3a_summary)[2]
h3a_median <- as.numeric(h3a_summary)[3]
h3a_3rdQ <- as.numeric(h3a_summary)[5]
c3b_summary <- summary(c3fmb[,1])
c3b_1stQ <- as.numeric(c3b_summary)[2]
c3b_median <- as.numeric(c3b_summary)[3]
```

```
c3b_3rdQ <- as.numeric(c3b_summary)[5]
h3b_summary <- summary(h3fmb[,1])
h3b_1stQ <- as.numeric(h3b_summary)[2]
h3b_median <- as.numeric(h3b_summary)[3]
h3b_3rdQ <- as.numeric(h3b_summary)[5]

#test 1
#judge whether the h3a_median is higher
  #(in theory, it is NOT for the valley marker)
if(h3a_median > c3a_median){
  h3a_HigherMedian <- 1
} else {
  h3a_HigherMedian <- 0
}

#judge whether the h3b_median is lower
  #(in theory, it is NOT for the valley marker)
if(h3b_median > c3b_median){
  h3b_HigherMedian <- 1
} else {
  h3b_HigherMedian <- 0
}

#test 2
#judge whether boxplot is separated and not overlapped
#when h3_median is higher than c3_median
if(c3a_3rdQ < h3a_1stQ){
  hih3_a_BoxSeparated <- 1
} else {
  hih3_a_BoxSeparated <- 0
}

if(c3a_3rdQ < h3a_1stQ){
  hih3_b_BoxSeparated <- 1
} else {
  hih3_b_BoxSeparated <- 0
```

```
}

#when h3_median is lower than c3_median
if(c3a_1stQ > h3a_3rdQ){
   loh3_a_BoxSeparated <- 1
} else {
   loh3_a_BoxSeparated <- 0
}

if(c3b_1stQ > h3b_3rdQ){
   loh3_b_BoxSeparated <- 1
} else {
   loh3_b_BoxSeparated <- 0
}

#test 3
#partial overlap of boxplot
#judge whether boxplot is more than 50%-separated
#test 3-1 and 3-2
#when h3_median is higher than c3_median
#test 3-1.
#median<1rdQ check
if(c3a_median < h3a_1stQ){
   hih3_c3a_med_under_h3a1stQ <- 1
} else {
   hih3_c3a_med_under_h3a1stQ <- 0
}

if(c3b_median < h3b_1stQ){
   hih3_c3b_med_under_h3b1stQ <- 1
} else {
   hih3_c3b_med_under_h3b1stQ <- 0
}

#test 3-2.
#3rdQ<median check
```

```
if(c3a_3rdQ < h3a_median){
  hih3_c3a3rdQ_under_h3a_med <- 1
} else {
  hih3_c3a3rdQ_under_h3a_med <- 0
}

if(c3b_3rdQ < h3b_median){
  hih3_c3b3rdQ_under_h3b_med <- 1
} else {
  hih3_c3b3rdQ_under_h3b_med <- 0
}

#test 3-3 and 3-4
#when h3_median is lower than c3_median
#test 3-3.
#median>3rdQ check
if(c3a_median > h3a_3rdQ){
  loh3_c3a_med_over_h3a3rdQ <- 1
} else {
  loh3_c3a_med_over_h3a3rdQ <- 0
}

if(c3b_median > h3b_3rdQ){
  loh3_c3b_med_over_h3b3rdQ <- 1
} else {
  loh3_c3b_med_over_h3b3rdQ <- 0
}

#test 3-4.
#1stQ>median check
if(c3a_1stQ > h3a_median){
  loh3_c3a1stQ_over_h3a_med <- 1
} else {
  loh3_c3a1stQ_over_h3a_med <- 0
}
```

```
if(c3b_1stQ > h3b_median){
  loh3_c3b1stQ_over_h3b_med <- 1
} else {
  loh3_c3b1stQ_over_h3b_med <- 0
}

#test 3 summary
#the xxh3_x_score_boxplot is a score that the two boxes in the plot is fully or partially separated.
  #the full score is 3.
hih3_a_score_boxplot <- hih3_a_BoxSeparated + hih3_c3a_med_under_h3a1stQ + hih3_c3a3rdQ_under_h3a_med
hih3_b_score_boxplot <- hih3_b_BoxSeparated + hih3_c3b_med_under_h3b1stQ + hih3_c3b3rdQ_under_h3b_med
loh3_a_score_boxplot <- loh3_a_BoxSeparated + loh3_c3a_med_over_h3a3rdQ + loh3_c3a1stQ_over_h3a_med
loh3_b_score_boxplot <- loh3_b_BoxSeparated + loh3_c3b_med_over_h3b3rdQ + loh3_c3b1stQ_over_h3b_med

#test 4
#sandwich status of the target between wn1 and wn2
#when it is, the fm value should be between 0 and 1
if(0 < c3a_median & c3a_median < 1){
  sand_c3a_median <- 1
} else {
  sand_c3a_median <- 0
}

if(0 < c3a_1stQ & c3a_1stQ < 1){
  sand_c3a_1stQ <- 1
} else {
  sand_c3a_1stQ <- 0
}

if(0 < h3a_median & h3a_median < 1){
  sand_h3a_median <- 1
```

```
} else {
  sand_h3a_median <- 0
}

if(0 < h3a_1stQ & h3a_1stQ < 1){
  sand_h3a_1stQ <- 1
} else {
  sand_h3a_1stQ <- 0
}

#summary of sandwich status
#the full mark is 4, but it is not the absolute requirement
sand_score_a <- sand_c3a_median + sand_c3a_1stQ + sand_h3a_median + sand_h3a_1stQ

if(0 < c3b_median & c3b_median < 1){
  sand_c3b_median <- 1
} else {
  sand_c3b_median <- 0
}

if(0 < c3b_1stQ & c3b_1stQ < 1){
  sand_c3b_1stQ <- 1
} else {
  sand_c3b_1stQ <- 0
}

if(0 < h3b_median & h3b_median < 1){
  sand_h3b_median <- 1
} else {
  sand_h3b_median <- 0
}

if(0 < h3b_1stQ & h3b_1stQ < 1){
  sand_h3b_1stQ <- 1
} else {
```

```
    sand_h3b_1stQ <- 0
  }

  sand_score_b <- sand_c3b_median + sand_c3b_1stQ + sand_h3b_median +
sand_h3b_1stQ

  #8: Calculate the p value by t-test
  a_ttest <- t.test(c3fma[,1], h3fma[,1], var.equal=T)
  p_a <- a_ttest$p.value

  b_ttest <- t.test(c3fmb[,1], h3fmb[,1], var.equal=T)
  p_b <- b_ttest$p.value

  #calculate the c3/h3 ratio
  a_h_c_ratio <- h3a_median/c3a_median
  b_h_c_ratio <- h3b_median/c3b_median

  #9: Record the results to the output dataframe
  tempo_anchor_results <- as.data.frame(t(c(wn1and2, wn1, wn2,
    h3a_HigherMedian, h3b_HigherMedian, a_h_c_ratio, b_h_c_ratio, p_a, p_b,
    hih3_a_score_boxplot, hih3_b_score_boxplot,
    loh3_a_score_boxplot, loh3_b_score_boxplot,
    sand_score_a, sand_score_b,
    hih3_a_BoxSeparated, hih3_c3a_med_under_h3a1stQ,
hih3_c3a3rdQ_under_h3a_med,
    hih3_b_BoxSeparated, hih3_c3b_med_under_h3b1stQ,
hih3_c3b3rdQ_under_h3b_med,
    loh3_a_BoxSeparated, loh3_c3a_med_over_h3a3rdQ, loh3_c3a1stQ_over_h3a_med,
    loh3_b_BoxSeparated, loh3_c3b_med_over_h3b3rdQ, loh3_c3b1stQ_over_h3b_med,
    sand_c3a_median, sand_c3a_1stQ, sand_h3a_median, sand_h3a_1stQ,
    sand_c3b_median, sand_c3b_1stQ, sand_h3b_median, sand_h3b_1stQ,
    c3a_1stQ, c3a_median, c3a_3rdQ, h3a_1stQ, h3a_median, h3a_3rdQ,
    c3b_1stQ, c3b_median, c3b_3rdQ, h3b_1stQ, h3b_median, h3b_3rdQ)))

    colnames(tempo_anchor_results) <- c("wn1and2", "wn1", "wn2",
      "h3a_HigherMedian", "h3b_HigherMedian", "a_h_c_ratio", "b_h_c_ratio", "p_a",
```

```
"p_b",
            "hih3_a_score_boxplot", "hih3_b_score_boxplot",
            "loh3_a_score_boxplot", "loh3_b_score_boxplot",
            "sand_score_a", "sand_score_b",
            "hih3_a_BoxSeparated", "hih3_c3a_med_under_h3a1stQ", "hih3_c3a3rdQ_under_h3a_med",
            "hih3_b_BoxSeparated", "hih3_c3b_med_under_h3b1stQ", "hih3_c3b3rdQ_under_h3b_med",
            "loh3_a_BoxSeparated", "loh3_c3a_med_over_h3a3rdQ", "loh3_c3a1stQ_over_h3a_med",
            "loh3_b_BoxSeparated", "loh3_c3b_med_over_h3b3rdQ", "loh3_c3b1stQ_over_h3b_med",
            "sand_c3a_median", "sand_c3a_1stQ", "sand_h3a_median", "sand_h3a_1stQ",
            "sand_c3b_median", "sand_c3b_1stQ", "sand_h3b_median", "sand_h3b_1stQ",
            "c3a_1stQ", "c3a_median", "c3a_3rdQ", "h3a_1stQ", "h3a_median", "h3a_3rdQ",
            "c3b_1stQ", "c3b_median", "c3b_3rdQ", "h3b_1stQ", "h3b_median", "h3b_3rdQ")

      #merge the generated data into output dataframe
      quest_anchor_summary = rbind(quest_anchor_summary, tempo_anchor_results)
      #10: Inward looping going out and iterate
  }
  #11: Outward looping going out and iterate
}

#12: Save the output dataframe
#write.csv(quest_anchor_summary,
#             "C:/users/akash/desktop/anchor_all_data.csv", row.names=FALSE)
filename_quest_anchor_summary <- paste(today2, "_a5_quest_anchor_alldata_", lda_peak_wn_string, ".csv", sep="")
filename2_quest_anchor_summary <- paste(pathinfo,"/", filename_quest_anchor_summary, sep="")
write.csv(quest_anchor_summary,
            filename2_quest_anchor_summary, row.names=FALSE)

#split the data into 4 category of high or low h3, and a- or b-basepoint
hih3_a_all_data <- dplyr::filter(quest_anchor_summary, h3a_HigherMedian==1)
```

```
hih3_b_all_data <- dplyr::filter(quest_anchor_summary, h3b_HigherMedian==1)
loh3_a_all_data <- dplyr::filter(quest_anchor_summary, h3a_HigherMedian==0)
loh3_b_all_data <- dplyr::filter(quest_anchor_summary, h3b_HigherMedian==0)

#select significant candidates
#select p<0.05
hih3_a_candidate <- dplyr::filter(hih3_a_all_data, as.numeric(p_a)<0.05)
hih3_b_candidate <- dplyr::filter(hih3_b_all_data, as.numeric(p_b)<0.05)
loh3_a_candidate <- dplyr::filter(loh3_a_all_data, as.numeric(p_a)<0.05)
loh3_b_candidate <- dplyr::filter(loh3_b_all_data, as.numeric(p_b)<0.05)

#select more than 1.5 fold absolute difference in ch_ratio
#anchor_candidate2 <- dplyr::filter(anchor_candidate1,
#                 a_ch_ratio>1.5|a_ch_ratio<0.75|b_ch_ratio>1.5|b_ch_ratio<0.75)

#convert the type of score_boxplot to numeric
hih3_a_candidate$hih3_a_score_boxplot                                          <-
as.numeric(hih3_a_candidate$hih3_a_score_boxplot)
hih3_b_candidate$hih3_b_score_boxplot                                          <-
as.numeric(hih3_b_candidate$hih3_b_score_boxplot)
loh3_a_candidate$loh3_a_score_boxplot                                          <-
as.numeric(loh3_a_candidate$loh3_a_score_boxplot)
loh3_b_candidate$loh3_b_score_boxplot                                          <-
as.numeric(loh3_b_candidate$loh3_b_score_boxplot)

#14: Sort them according to the ranking
#sorting
#if you wish to sort in the descending order, replace "p" to "desc(p)"
hih3_a_candidate2 <- arrange(hih3_a_candidate, desc(hih3_a_score_boxplot))
hih3_b_candidate2 <- arrange(hih3_b_candidate, desc(hih3_b_score_boxplot))
loh3_a_candidate2 <- arrange(loh3_a_candidate, desc(loh3_a_score_boxplot))
loh3_b_candidate2 <- arrange(loh3_b_candidate, desc(loh3_b_score_boxplot))

#eliminate unnecessary columns for the output
hih3_a_candidate3 <- dplyr::select(hih3_a_candidate2, wn1and2, wn1, wn2,
  h3a_HigherMedian, a_h_c_ratio, p_a, hih3_a_score_boxplot, sand_score_a,
```

```
    hih3_a_BoxSeparated, hih3_c3a_med_under_h3a1stQ, hih3_c3a3rdQ_under_h3a_med,
    sand_c3a_median, sand_c3a_1stQ, sand_h3a_median, sand_h3a_1stQ,
    c3a_1stQ, c3a_median, c3a_3rdQ, h3a_1stQ, h3a_median, h3a_3rdQ)

hih3_b_candidate3 <- dplyr::select(hih3_b_candidate2, wn1and2, wn1, wn2,
    h3b_HigherMedian, b_h_c_ratio, p_b, hih3_b_score_boxplot, sand_score_b,
    hih3_b_BoxSeparated, hih3_c3b_med_under_h3b1stQ, hih3_c3b3rdQ_under_h3b_med,
    sand_c3b_median, sand_c3b_1stQ, sand_h3b_median, sand_h3b_1stQ,
    c3b_1stQ, c3b_median, c3b_3rdQ, h3b_1stQ, h3b_median, h3b_3rdQ)

loh3_a_candidate3 <- dplyr::select(loh3_a_candidate2, wn1and2, wn1, wn2,
    h3a_HigherMedian, a_h_c_ratio, p_a, loh3_a_score_boxplot, sand_score_a,
    loh3_a_BoxSeparated, loh3_c3a_med_over_h3a3rdQ, loh3_c3a1stQ_over_h3a_med,
    sand_c3a_median, sand_c3a_1stQ, sand_h3a_median, sand_h3a_1stQ,
    c3a_1stQ, c3a_median, c3a_3rdQ, h3a_1stQ, h3a_median, h3a_3rdQ)

loh3_b_candidate3 <- dplyr::select(loh3_b_candidate2, wn1and2, wn1, wn2,
    h3b_HigherMedian, b_h_c_ratio, p_b, loh3_b_score_boxplot, sand_score_b,
    loh3_b_BoxSeparated, loh3_c3b_med_over_h3b3rdQ, loh3_c3b1stQ_over_h3b_med,
    sand_c3b_median, sand_c3b_1stQ, sand_h3b_median, sand_h3b_1stQ,
    c3b_1stQ, c3b_median, c3b_3rdQ, h3b_1stQ, h3b_median, h3b_3rdQ)

#14: Save the output dataframe
#write.csv(hih3_a_candidate3,
#    "C:/users/akash/desktop/hih3_a_candidate3.csv", row.names=FALSE)
filename_hih3_a_candidate <- paste(today2, "_a5_hih3_a_candidatefm_",
lda_peak_wn_string, ".csv", sep="")
filename2_hih3_a_candidate <- paste(pathinfo,"/", filename_hih3_a_candidate, sep="")
write.csv(hih3_a_candidate3,
            filename2_hih3_a_candidate, row.names=FALSE)

#write.csv(hih3_b_candidate3,
#    "C:/users/akash/desktop/hih3_b_candidate3.csv", row.names=FALSE)
filename_hih3_b_candidate <- paste(today2, "_a5_hih3_b_candidatefm_",
lda_peak_wn_string, ".csv", sep="")
filename2_hih3_b_candidate <- paste(pathinfo,"/", filename_hih3_b_candidate, sep="")
```

```
write.csv(hih3_b_candidate3,
          filename2_hih3_b_candidate, row.names=FALSE)

#write.csv(loh3_a_candidate3,
#   "C:/users/akash/desktop/loh3_a_candidate3.csv", row.names=FALSE)
filename_loh3_a_candidate <- paste(today2, "_a5_loh3_a_candidatefm_", lda_peak_wn_string, ".csv", sep="")
filename2_loh3_a_candidate <- paste(pathinfo,"/", filename_loh3_a_candidate, sep="")
write.csv(loh3_a_candidate3,
          filename2_loh3_a_candidate, row.names=FALSE)

#write.csv(loh3_b_candidate3,
#   "C:/users/akash/desktop/loh3_b_candidate3.csv", row.names=FALSE)
filename_loh3_b_candidate <- paste(today2, "_a5_loh3_b_candidatefm_", lda_peak_wn_string, ".csv", sep="")
filename2_loh3_b_candidate <- paste(pathinfo,"/", filename_loh3_b_candidate, sep="")
write.csv(loh3_b_candidate3,
          filename2_loh3_b_candidate, row.names=FALSE)
#End of script
```

Script code 5: Evaluation of Fm-anchor candidates

```
#salma_a6_anchor_candi_evalu_211225.r
#anchor candidate evaluation
  #for selecting the most effective pair of anchor points
  #for the potential ftir markers
#this is for Salma's data on N61 c3-h3 chamber comparison.

#clear the brain
rm(list=ls())

#library to register
#ggplot2 and dplyr are in tidyverse
library(conflicted)
library(tidyverse)
library(MASS)
```

```
library(klaR)
library(caret)

#obtain date information
today <- Sys.Date()
yr <- substr(today, 3,4)
mo <- substr(today, 6,7)
day <- substr(today, 9,10)
today2 <- paste(yr, mo, day, sep="")

#obtain desktop folder information for a windows user
#you must change the string within "xxx" below according to your computer
desktopfolder <- "akash"

#assemble path info
pathinfo <- paste("C:/users/",desktopfolder,"/desktop/", sep="")

#import the Peak/Valley CandidateXXXX.csv"
print("Please select xxxxxx_a4_PeakCandidateXXXX.csv file")
print("Or, please select xxxxxx_a4_ValleyCandidateXXXX.csv file")
print("Please make sure that the cols 7 and 8 for search boundary is filled")
print("Typically, set the value 150 larger and smaller than the target")
print("Please select xxxxxx_a5_PeakCandidateXXXX.csv file")
print("Or, please select xxxxxx_a5_ValleyCandidateXXXX.csv file")
PeakCandidateInput <- file.choose()
PeakCandidateInput2 <- read.csv(PeakCandidateInput,
                                header = T)

#extract LDA-peak and boundary information
lda_peak_wn <- PeakCandidateInput2[1,1]
lda_LargerWnBoundary_wn <- PeakCandidateInput2[1,7]
lda_SmallerWnBoundary_wn <- PeakCandidateInput2[1,8]

#modify peak_wn variable to the style of column name
lda_peak_wn_string <- as.character(lda_peak_wn)
lda_peak_wn_colname <- paste("X",lda_peak_wn_string, sep="")
```

```
#import the 2nd "xxxxxx_a5_xxh3_x_candidatefmxxxx.csv" data
print("Please select xxxxxx_a5_xxh3_x_candidatefmXXXX.csv file")
candi1 <- file.choose()
candi2 <- read.csv(candi1,
                   header = T)

#import the 3rd "xxxxxx_a1_specmean.csv" data
print("Please select xxxxxx_a1_specmean.csv file")
spec1 <- file.choose()
twospec <- read.csv(spec1,
                    header = T)

#arrange the spectra
twospec2 <- dplyr::select(twospec, -(c(1:1)))
colnames(twospec2) <- seq(from=4000, to=400, by=-1)

wnlist1 <- as.data.frame(t(seq(from=4000, to=400, by=-1)))
colnames(wnlist1) <- seq(from=4000, to=400, by=-1)
twospec3 <- rbind(wnlist1, twospec2)

longspec3 <- as.data.frame(t(twospec3))
colnames(longspec3) <- c("wn", "c3", "h3")

dev.new()
ggplot(longspec3, aes(x = wn ,y = c3)) +
geom_point(size=0.3)
theme.bw()

#narrow down the candidate
#according to the number of candidate,
#mask/unmask the filtering with scores
names(candi2)[7] <- "score_boxplot"
names(candi2)[8] <- "sand_score"
candi4 <- dplyr::filter(candi2, score_boxplot==3)
#candi4 <- dplyr::filter(candi4, sand_score ==4)
```

```
candi4$wn1 <- as.numeric(substr(candi4$wn1,2,5))
candi4$wn2 <- as.numeric(substr(candi4$wn2,2,5))

#trim down the spectral data
cutsite1 <- 4002 - lda_SmallerWnBoundary_wn
cutsite2 <- 4000 - lda_LargerWnBoundary_wn
spec4 <- dplyr::select(twospec3, -c(cutsite1:3601))
spec4 <- dplyr::select(spec4, -c(1:cutsite2))
longspec4 <- as.data.frame(t(spec4))

colnames(longspec4) <- c("wn", "c3", "h3")
longspec4 <- transform(longspec4, target=0)
longspec4 <- transform(longspec4, scoreL=0)
longspec4 <- transform(longspec4, scoreS=0)
longspec4 <- transform(longspec4, lower_abs_anchor=0)
longspec4 <- transform(longspec4, higher_abs_anchor=0)
longspec4 <- transform(longspec4, suffix=NA)

#normalize the spectra
longspec4$c3 <- (longspec4$c3-min(longspec4$c3))/(max(longspec4$c3)-min(longspec4$c3))
longspec4$h3 <- (longspec4$h3-min(longspec4$h3))/(max(longspec4$h3)-min(longspec4$h3))

#mark the target wavenumber
#mark the hih3_a_score_boxplot
nrow_longspec4 <- nrow(longspec4)
nrow_candi4 <- nrow(candi4)
for (i in 1:nrow_longspec4){
  if(longspec4[i,1]==lda_peak_wn){
    longspec4[i,4] <- 0.1
  }

  for (j in 1:nrow_candi4){
    temp_wn1 <- candi4[j,2]
    temp_wn2 <- candi4[j,3]
```

```
      if(longspec4[i,1]==temp_wn1){
        longspec4[i,5] <- longspec4[i,5]+0.01
      }
      if(longspec4[i,1]==temp_wn2){
        longspec4[i,6] <- longspec4[i,6]+0.01
      }
  }
}
#save the longspec4 as csv
filename_specanchor <- paste(today2, "_a6_", "specanchor_", lda_peak_wn_string, ".csv",
sep="")
filename2_specanchor <- paste(pathinfo,"/", filename_specanchor, sep="")
write.csv(longspec4,
          filename2_specanchor, row.names=FALSE)

#draw the anchor points
dev.new()
plotanchor4 <- ggplot(longspec4) +
  theme_light()+
  geom_line(aes(x=wn, y=c3),
            colour="deepskyblue", size=0.3)+
  geom_line(aes(x=wn, y=h3),
            colour="salmon", size=0.3)+
  geom_line(aes(x=wn, y=target),
           colour="salmon", size=0.3)+
  geom_line(aes(x=wn, y=scoreL),
            colour="black", size=0.3)+
  geom_line(aes(x=wn, y=scoreS),
            colour="black", size=0.3)
print(plotanchor4)

#save the plot as png format(you can change to .jpeg, .tiff, etc)
#unit is in inch

filename_plotanchor4 <- paste(today2, "_a6_", "plotanchor4.png", sep="")
filename2_plotanchor4 <- paste(pathinfo,"/", filename_plotanchor4, sep="")
```

```
ggsave(file = filename2_plotanchor4,
           plot = plotanchor4, dpi = 100,
           width = 7.2, height = 4.8)
#End of script
```

Script code 6: Boxplot analysis of Fm markers

```
#salma_a7_ftir_marker_boxplot_211225a.r
#a7_ftir marker boxplot
#for salma's 1st paper

#clear the brain
rm(list=ls())

#library to register
#ggplot2 and dplyr are in tidyverse
library(conflicted)
library(tidyverse)
library(MASS)
library(klaR)
library(caret)

#obtain date information
today <- Sys.Date()
yr <- substr(today, 3,4)
mo <- substr(today, 6,7)
day <- substr(today, 9,10)
today2 <- paste(yr, mo, day, sep="")

#obtain desktop folder information for a windows user
#you must change the string within "xxx" below according to your computer
desktopfolder <- "akash"

#assemble path info
pathinfo <- paste("C:/users/",desktopfolder,"/desktop/", sep="")
```

```
#import the 1st, compiled ftir csv data
print("Please specify xxxxxx_a2_specpile_processed.csv")
specpile1 <- file.choose()
specpile2 <- read.csv(specpile1,
                      header = T)

#change the variable types of "condition" and "genotype"
#to factor format
specpile2$condition <- factor(specpile2$condition)
specpile2$genotype <- factor(specpile2$genotype)

#import the "a6_specanchor_xxxx_v2.csv" file
  #integer of "1" should be input at col 7 and 8
print("Please specify a6_xxxxxx_specanchor_xxxx_v2.csv")
specanchor1 <- file.choose()
specanchor2 <- read.csv(specanchor1,
                        header = T)

#extract wavenumber info for target and anchors
n_specanchor2 <- nrow(specanchor2)
for(i in 1:n_specanchor2){
  if(specanchor2[i,4]==0.1){
    wn_target <- specanchor2[i,1]
  }
  if(specanchor2[i,7]==1){
    wn_lower_abs_anchor <- specanchor2[i,1]
  }
  if(specanchor2[i,8]==1){
    wn_higher_abs_anchor <- specanchor2[i,1]
    suffix_ancpair <- specanchor2[i,9]
  }
}
wn_target_col <- 4005-wn_target
wn_lower_anchor_col <- 4005-wn_lower_abs_anchor
wn_higher_anchor_col <- 4005-wn_higher_abs_anchor
```

```
wn_target_chr <- as.character(wn_target)

#calculate fm value
specpile3 <- mutate(specpile2, fm_numerator=(specpile2[,wn_target_col] - specpile2[,wn_lower_anchor_col]))
specpile3 <- mutate(specpile3, fm_denominator=(specpile2[,wn_higher_anchor_col] - specpile2[,wn_lower_anchor_col]))
specpile3 <- mutate(specpile3, fm=(fm_numerator/fm_denominator))
specpile3 <- mutate(specpile3, target_abs=specpile2[,wn_target_col])
specpile3 <- mutate(specpile3, lower_abs_anchor_abs=specpile2[,wn_lower_anchor_col])
specpile3 <- mutate(specpile3, higher_abs_anchor_abs=specpile2[,wn_higher_anchor_col])

specfm1 <- dplyr::select(specpile3, c(1, 2, 3606, 3607, 3608, 3609, 3610, 3611))

#save the fm info as csv
filename_specfm1 <- paste(today2, "_a7_specfm1_", wn_target_chr, suffix_ancpair, ".csv", sep="")
filename2_specfm1 <- paste(pathinfo,"/", filename_specfm1, sep="")
write.csv(specfm1,
          filename2_specfm1, row.names=FALSE)

#save the second cv
specpile4 <- dplyr::select(specpile2, c(wn_target_col, wn_higher_anchor_col, wn_lower_anchor_col))
filename_specpile4 <- paste(today2, "_a7_specpile4_", wn_target_chr, suffix_ancpair, ".csv", sep="")
filename2_specpile4 <- paste(pathinfo,"/", filename_specpile4, sep="")
write.csv(specpile4,
          filename2_specpile4, row.names=FALSE)

#Reverse version
#calculate fm value for reverse version
specpile3rev <- mutate(specpile2, fm_numerator=(specpile2[,wn_target_col] - specpile2[,wn_higher_anchor_col]))
specpile3rev <- mutate(specpile3rev, fm_denominator=(specpile2[,wn_lower_anchor_col] - specpile2[,wn_higher_anchor_col]))
```

```
specpile3rev <- mutate(specpile3rev, fm=(fm_numerator/fm_denominator))
specpile3rev <- mutate(specpile3rev, target_abs=specpile2[,wn_target_col])
specpile3rev <- mutate(specpile3rev,
lower_abs_anchor_abs=specpile2[,wn_lower_anchor_col])
specpile3rev <- mutate(specpile3rev,
higher_abs_anchor_abs=specpile2[,wn_higher_anchor_col])

specfm1rev <- dplyr::select(specpile3rev, c(1, 2, 3606, 3607, 3608, 3609, 3610, 3611))

#save the fm info as csv
filename_specfm1rev <- paste(today2, "_a7_specfm1rev_", wn_target_chr, suffix_ancpair,
".csv", sep="")
filename2_specfm1rev <- paste(pathinfo,"/", filename_specfm1rev, sep="")
write.csv(specfm1rev,
          filename2_specfm1rev, row.names=FALSE)

#save the second cv
specpile4rev <- dplyr::select(specpile2, c(wn_target_col, wn_higher_anchor_col,
wn_lower_anchor_col))
filename_specpile4rev <- paste(today2, "_a7_specpile4rev_", wn_target_chr, suffix_ancpair,
".csv", sep="")
filename2_specpile4rev <- paste(pathinfo,"/", filename_specpile4rev, sep="")
write.csv(specpile4rev,
          filename2_specpile4rev, row.names=FALSE)

#make a boxplot
#in the following, "x" should be the grouping variable,
#usually in the category variable, such as condition
#"y" should be numerical variable such as fm.
#xlab("xxx") is for the label of figure
#for color pallet, check the following
# http://sape.inf.usi.ch/quick-reference/ggplot2/colour
wn_target_label <- paste("fm", wn_target_chr, suffix_ancpair, sep="")
dev.new()
fm_boxplot <- ggplot(specfm1, aes(x = condition, y = fm, fill=condition)) +
  stat_boxplot(geom = "errorbar", width = 0.3)+
```

```
  geom_boxplot(outlier.size=1) +
  scale_fill_manual(values=c("deepskyblue", "salmon")) +
  #   geom_point(size=0.3, color='lightgray', alpha=0.5) +
  xlab("Condition") +
  ylab(wn_target_label) +
  #if you change the range of y-axis, use the follow line
  #   ylim(-20, 20)+
  theme_bw()
print(fm_boxplot)

#save the same fm_boxplot as png file in the desktop
filename_fm_boxplot <- paste(today2, "_a7_", wn_target_label, "_boxplot.png", sep="")
filename2_fm_boxplot <- paste(pathinfo,"/",   filename_fm_boxplot, sep="")
ggsave(file = filename2_fm_boxplot,
       plot = fm_boxplot, dpi = 100,
       width = 2.4, height = 2.4)

#reverse version
wn_target_label_rev <- paste("fm", wn_target_chr, suffix_ancpair, "_rev", sep="")
dev.new()
fmrev_boxplot <- ggplot(specfm1rev, aes(x = condition, y = fm, fill=condition)) +
  stat_boxplot(geom = "errorbar", width = 0.3)+
  geom_boxplot(outlier.size=1) +
  scale_fill_manual(values=c("deepskyblue", "salmon")) +
  #   geom_point(size=0.3, color='lightgray', alpha=0.5) +
  xlab("Condition") +
  ylab(wn_target_label_rev) +
  #if you change the range of y-axis, use the follow line
  #   ylim(-20, 20)+
  theme_bw()
print(fmrev_boxplot)

#save the same fm_boxplot as png file in the desktop
filename_fmrev_boxplot <- paste(today2, "_a7_", wn_target_label_rev, "_boxplot.png",
sep="")
filename2_fmrev_boxplot <- paste(pathinfo,"/",   filename_fmrev_boxplot, sep="")
```

```
ggsave(file = filename2_fmrev_boxplot,
        plot = fmrev_boxplot, dpi = 100,
        width = 2.4, height = 2.4)
#End of script
```
