# Supplementary figures and images for: Chemical Fingerprinting of Heat Stress Responses in the Leaves of Common Wheat by Fourier Transform Infrared Spectroscopy
Source: Int J Mol Sci. 2022 Mar 4;23(5):2842. doi: 10.3390/ijms23052842 (PMC8911002; doi:10.3390/ijms23052842)

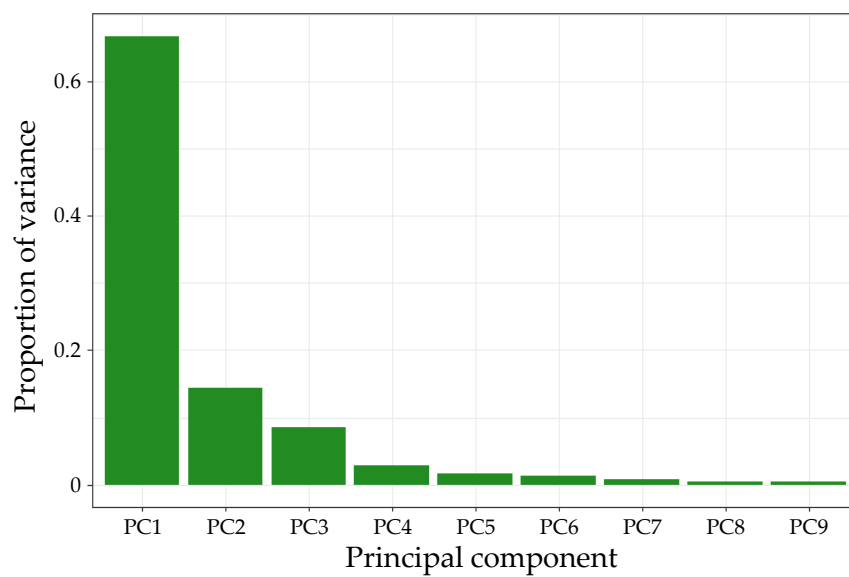

Figure S1. Variance explained by the first 9 components in principal component analysis.

Supplement: Supplementary file 1 [file ijms-23-02842-s001.zip › Supplementary Figure S1.pdf]
